# Symptomatic IgG4-Related Prostatitis Simultaneously Diagnosed with Aggressive Prostate Cancer

**DOI:** 10.1155/2020/6045328

**Published:** 2020-03-03

**Authors:** Taisuke Ezaki, Seiya Akatsuka, Tansei Sanjo, Takeshi Masuda

**Affiliations:** ^1^Department of Urology, Saitama City Hospital, Saitama, Japan; ^2^Division of Pathology, Department of Central Laboratory, Saitama City Hospital, Saitama, Japan

## Abstract

IgG4-related disease is a systemic fibroinflammatory disorder that occasionally affects the prostate. It is usually considered that patients with IgG4-related disease are at high risk of developing malignancies. A 71-year-old man presented to our hospital with a chief complaint of urinary retention. Prostate biopsy revealed concomitant IgG4-related prostatitis and prostate cancer. IgG4-related prostatitis was a possible cause of urinary retention, and the aggressive nature of prostate cancer was the cause of the patient's death 2 years after diagnosis. This is the fourth case report of prostate cancer accompanied by IgG4-related prostatitis; however, there have been no reports of the two diseases coexisting with high clinical significance. Our case report indicates that patients diagnosed with IgG4-related prostatitis should be carefully followed up considering the risk of prostate cancer.

## 1. Introduction

IgG4-related disease is a rare systemic fibroinflammatory disorder characterized by the enlargement of affected organs, a significant increase in serum IgG4 levels, tissue infiltration with IgG4-positive plasma cells, and fibrosis [1–3]. IgG4-related disease of the genitourinary tract is relatively rare; however, it occasionally affects the prostate [4–8].

Although a complete consensus has not yet been established, patients with IgG4-related disease are typically considered at high risk of developing malignant disease [9–12]. Here, we report a case of prostate cancer accompanied by IgG4-related prostatitis.

## 2. Case Presentation

**Figure 1:**
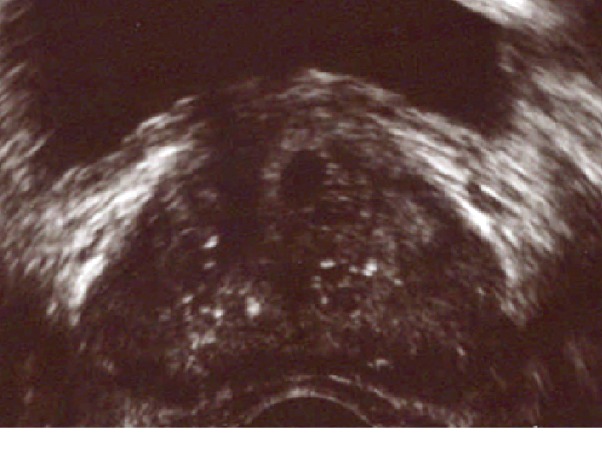
Image obtained using transrectal ultrasound at the second biopsy showing an enlarged prostate (axial view). The estimated prostate volume was 49 cm^3^. Magnetic resonance imaging was not performed because the patient had undergone femoral head replacement.

**Figure 2:**
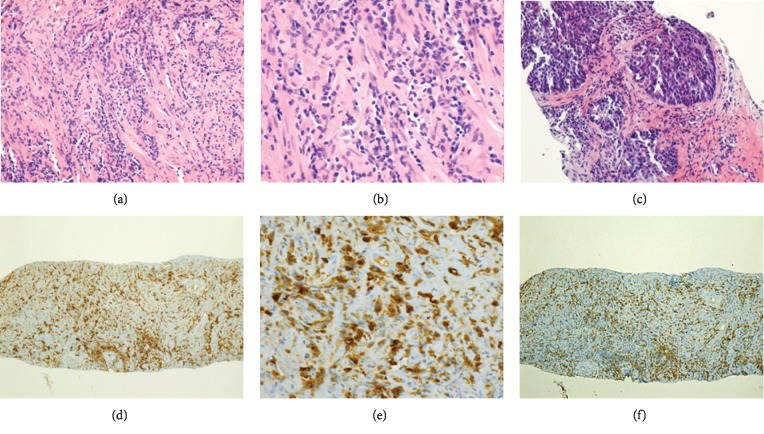
Paraffin section of prostate biopsy tissue. (a–c) Hematoxylin and eosin (H&E) staining and immunohistochemical staining for (d, e) IgG4 and (f) IgG. Original magnification: (d, e) ×100; (a, c) ×200; and (b, e) ×400. H&E staining shows storiform fibrosis, exocrine glands with periglandular fibrosis, and lymphoplasmacytic and eosinophilic infiltration (a, b). Adenocarcinoma, Gleason score 5 + 4, was found in 1/12 needle cores, which occupied 10% of the core area, at the second biopsy (c). Indirect immunohistochemical staining for IgG4 revealed multiple IgG4-positive plasma cells (>50/high-power field (HPF)) infiltrating the prostate (d, e). The ratio of IgG4-positive/IgG-positive plasma cells was >50% (the histopathological diagnostic criteria for IgG4-related disease included marked lymphoplasmacytic infiltration, fibrosis, IgG4 positivity in >40% of IgG-positive plasma cells, and >10 IgG4-positive plasma cells/HPF of a biopsy sample).

A 71-year-old Japanese man presented to our hospital with a chief complaint of urinary retention. He had chronic obstructive pulmonary disease, hypertension, and a history of left femoral head replacement due to osteoarthritis of the hip. Additionally, he had a history of alcohol consumption and cigarette smoking. Prostate volume of 42 cm^3^ was defined using transabdominal ultrasonography. Urinary catheterization was performed, and alpha-blocker treatment was initiated. A follow-up examination conducted 1 month later showed serum prostate-specific antigen (PSA) levels of 74 ng/mL, although the last PSA test performed a year ago had revealed normal PSA levels. Blood and urine test results were nearly within normal limits, except that mild anemia and mild hypoalbuminemia were noted. Prostate tissue samples were obtained via transrectal needle biopsy. Digital rectal examination revealed a firm prostate; however, a prostate tissue sample stained with hematoxylin and eosin showed inflammation but no malignant cells. One month after the first biopsy, urinary retention recurred, but 2 weeks of clean intermittent self-catheterization improved the obstructive symptoms. One month later, the patient had elevated PSA levels of 180 ng/mL, and a second prostate biopsy was performed Figure 1. Adenocarcinoma (Gleason score 5 + 4) was detected in 1/12 needle cores Figure 2(c). Computed tomography revealed pelvic lymph node swelling, and multiple bone metastases (lumbar spine, pubic bone, and left acetabulum) were detected using bone scintigraphy; therefore, the patient was diagnosed with stage 4 prostate cancer. Moreover, we detected abundant plasma cell infiltration with periglandular storiform fibrosis in prostate tissue samples Figures 2(a) and 2(b). Although plasma cell clonality was not detected, IgG4 immunochemical staining demonstrated IgG4-positive plasma cells Figures 2(d) and 2(e). The ratio of IgG4-positive/IgG-positive plasma cells was >50% Figure 2(f). Given the result of the second biopsy, the initial prostate biopsies were reviewed again, and large numbers of IgG4-positive plasma cells were observed. At this point, the serum IgG4 levels were 1310 mg/dL (normal: <117 mg/dL), and a diagnosis of simultaneous IgG4-related prostatitis and prostate cancer was made. The patient immediately received combined androgen blockade treatment, and his serum PSA levels decreased to 1.7 ng/mL for 4 months. However, he developed castration resistance after 8 months of hormone therapy. Although abiraterone, docetaxel, and cabazitaxel were administered, cancer progressed, and multiple liver metastases appeared. He died 24 months after the diagnosis of prostate cancer. No treatment was given for IgG4-related prostatitis as the patient could urinate without a catheter at the time of diagnosis confirmation. The serum IgG4 levels slightly decreased to 883 mg/dL in the 7^th^ month of hormonal therapy and returned to normal when prednisolone at a dose of 10 mg/day was coadministered with abiraterone in the following month. Notably, he never complained of urinary retention until death.

## 3. Discussion

IgG4-related prostatitis was first reported by Yoshimura et al. [4], and subsequently, additional studies were conducted on the disease [5–8]. In patients with IgG4-related prostatitis, histological examination of the prostate reveals severe atrophy of the exocrine glands, dense fibrosis, and abundant infiltration of lymphocytes and IgG4-positive plasma cells [4]. IgG4-related prostatitis is occasionally accompanied by IgG4-related disease in other organs [4–8]. Such patients may experience resolution of the urinary symptoms after steroid treatment [5, 6, 8] and exhibit varying levels of serum PSA ranging from 0.1 to 54.0 ng/mL [6–8].

In the present case, acute urinary retention resulted in a hospital visit. Buijs et al. reported that >50% of patients with IgG4-related prostatitis presented with obstructive urinary tract symptoms [8], and we believe that acute inflammation due to IgG4-related prostatitis played an important role in causing urinary retention in our patient. Although prostate cancer can obstruct urine flow in the absence of inflammation, we believe that concomitant inflammation increased the severity of the obstructive symptoms. The observation that the urinary symptoms were ameliorated before cancer treatment suggested that prostate cancer was not the sole cause of urinary retention. As the patient's urinary symptoms were manageable at the time of diagnosis, we did not administer steroid therapy, although steroids have been used to effectively treat the urinary symptoms of IgG4-related prostatitis in previous case reports [5, 6, 8]. Usually, treatment of IgG4-related disease is initiated with 40 mg prednisolone or at a dose of 0.6 mg/kg of body weight/day [2], but spontaneous remission has also been reported [13]. Further, wait-and-see management may be more appropriate in some patients, given the side effects of steroids. In the present case, our strategy appeared reasonable as there was no recurrence of urinary retention.

To the best of our knowledge, only three cases of prostate cancer with IgG4-related prostatitis have been reported to date [6, 14]. The initial serum PSA levels of all three patients ranged from 5.5 to 8.2 ng/mL; two of them underwent radical prostatectomy. Although the case report on one patient did not clearly document prostate cancer treatment, PSA levels were suggestive of nonadvanced cancer. As regards urinary symptoms, symptomatic prostatitis was reported in one patient who presented with dysuria, whereas the other two reports did not provide details on symptoms involving the lower urinary tract. Therefore, this is the fourth case report of prostate cancer with IgG4-related prostatitis. However, inflammation caused by IgG4-related prostatitis was sufficiently severe to cause urinary retention, and prostate cancer was aggressive and life-threatening. No reports have demonstrated the coexistence of two diseases with high clinical significance. Therefore, we consider this case unique and worth reporting.

In our case report, the histopathological characteristics of IgG4-related prostatitis were retrospectively detected in the specimens of the first biopsy. As the patient had no history of IgG4-related disease, the immediate diagnosis of IgG4-related prostatitis was difficult. Nevertheless, an additional careful examination could have led to the diagnosis of IgG4-related prostatitis after the first biopsy. However, if steroid therapy administered for IgG4-related prostatitis had reduced the serum PSA levels, the detection of prostate cancer would have been delayed. Our case report indicates that patients diagnosed with IgG4-related prostatitis based on the symptoms of inflammation should be carefully followed up considering the risk of prostate cancer.

In the present case report, the association between IgG4-related prostatitis and the development of prostate cancer remains unclear. However, a possibility exists that IgG4-related prostatitis and prostate cancer, which manifested concomitantly and were diagnosed almost simultaneously, were associated and that their concomitant existence was not a coincidence. However, further investigation is necessary to ascertain the association between these two diseases.
